# Une angiocholite secondaire à un thrombus tumoral d'une tumeur neuroendocrine primitive du foie

**DOI:** 10.11604/pamj.2015.22.308.8185

**Published:** 2015-11-26

**Authors:** Hicham Baba, Mohamed Allaoui, Mohammed Elfahssi, Ahmed Bounaim, Abdelmounaim Ait Ali, Mohamed Oukabli, Khalid Sair, Aziz Zentar

**Affiliations:** 1 Service de Chirurgie Viscérale, Hôpital Militaire d'Instruction Mohammed V, Rabat, Maroc; 2 Service d'Anatomie Pathologique, Hôpital Militaire d'Instruction Mohammed V, Rabat, Maroc

**Keywords:** Angiocholite, tumeur neuroendocrine, foie, cholangitis, neuroendocrine tumor, liver

## Abstract

Nous rapportons le cas exceptionnel d'une patiente de 54 ans prise en charge pour une angiocholite due à un thrombus tumoral, d'une tumeur neuroendocrine primitive (TNE I^ve^) du foie, dans la voie biliaire principale.

## Introduction

Les tumeurs neuroendocrines digestives sont rares, moins de 1% des tumeurs malignes, et prennent leur origine dans le tube digestif dans 67,5% des cas [1]. Les TNE I^ves^ du foie sont exceptionnelles, en effet, moins d'une centaine de cas ont été décrits dans la littérature depuis le premier cas rapporté par EDMONDSON en 1958 [2, 3].

## Patient et observation

**Figure 1:**
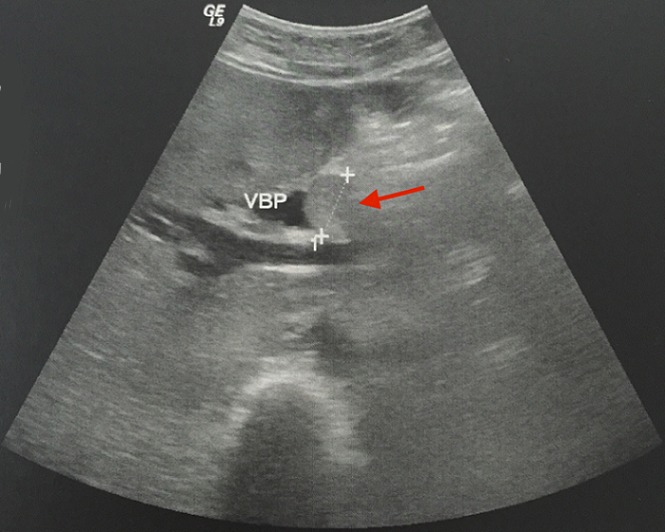
Echographie: bourgeon tumoral dans la VBP (flèche rouge)

**Figure 2:**
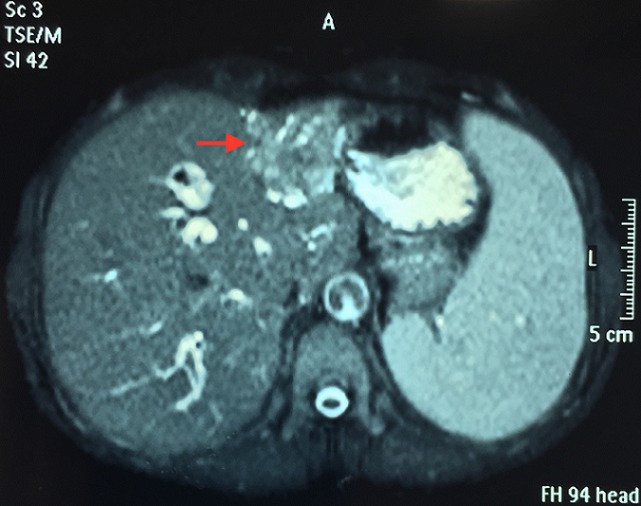
IRM: atrophie du foie gauche, lésion mal limitée (flèche)

**Figure 3:**
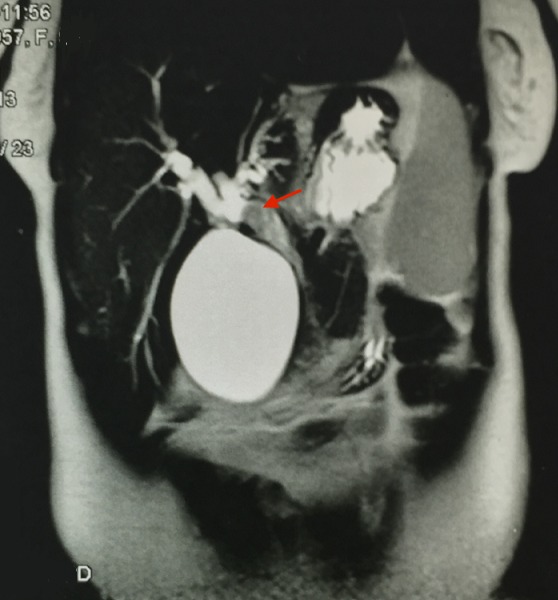
IRM: bourgeon tumoral dans la VBP (flèche)

**Figure 4:**
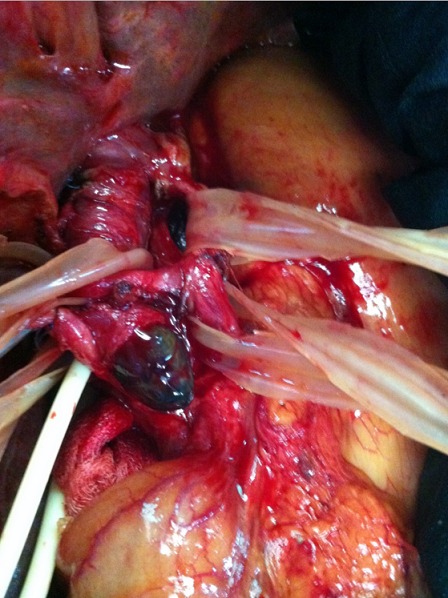
Vue opératoire: issue du thrombus tumoral par la cholédocotomie

**Figure 5:**
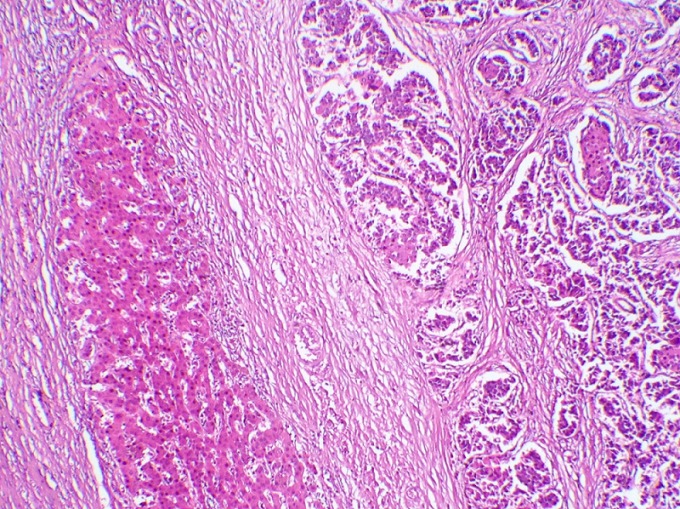
Parenchyme hépatique infiltré par une prolifération tumorale carcinomateuse, faite de nids, de massifs et parfois de cordons (HE, Gx50)

Mme N.N, 58 ans, ayant comme antécédent une thyroïdectomie totale pour un goitre multihétéronodulaire, présentait 4 mois avant son admission des coliques hépatiques, un ictère choléstatique d'installation progressive avec prurit et des accès fébriles. L'examen clinique trouvait une patiente en assez bon état général, avec un ictère cutanéo-muqueux franc et des lésions de grattages au niveau des membres et du tronc. L'examen abdominal notait une grosse vésicule biliaire palpable. Le reste de l'examen somatique était sans particularité. L’échographie abdominale avait montrait une dilatation des voies biliaires intrahépatiques en amont d'un processus tissulaire de la voie biliaire principale (VBP) et une vésicule biliaire (VB) distendue (Figure 1). La BILI IRM avait objectivé une atrophie du foie gauche siège d'une lésion mal limitée hypo intense en T1 et hyper intense en T2, une dilatation des VBIH avec présence d'un bourgeon tissulaire, mesurant 20 mm de diamètre, au niveau des branches gauches, iso intense en T1 et T2 et qui se réhaussait après injection du PC au temps tardif; ce bourgeon se prolongeait au niveau de la partie proximale de la VBP, englobait une lithiase de 14 mm de diamètre, et obstruait le canal cystique (Figure 2, Figure 3). Le bilan biologique montrait: Bilirubine totale 138 mg/l, ALAT 110 UI/l, ASAT 99UI/l, PAL 403 UI/l, GGT 117UI/l. une ponction-biopsie du foie, au niveau de la lésion, avait été réalisée dont l’étude histologique avait noté une cirrhose biliaire primitive sans signes de malignité. La patiente était opérée, l'exploration notait un foie de cholestase avec une lésion ombiliquée du foie gauche, une VB distendue et une VBP dilatée dont la palpation permettait de percevoir un matériel intra luminal. Le reste de l'exploration abdominale ne montrait pas de lésion suspecte. Une cholécystectomie était réalisé, la section de la VBP au dessous de l'abouchement du canal cystique donnait issue à un thrombus tumoral avec une paroi biliaire souple et macroscopiquement saine (Figure 4), une hépatectomie gauche était réalisée emportant la VBP, le rétablissement de la continuité biliaire était assuré par une anastomose du canal hépatique droit sur une anse en Y. Les suites opératoires étaient simples et la patiente est sortie à J 6 post opératoire. L’étude histologique de la pièce opératoire montrait une prolifération carcinomateuse faite essentiellement d'amas, de nids et de massifs avec présence par endroit de rosettes, les cellules tumorales avaient un cytoplasme éosinophile aux noyaux assez monomorphes avec une chromatine d'aspect « sel et poivre », les figures mitotiques étaient rares (1mitose/10 champs au grossissement X 40), le complément immunohistochimique montrait une positivité des cellules tumorales pour la chromogranine, la synaptophysine et la CK 19 et le marquage par le Ki67 était estimé à 15%, le bourgeon tumoral intracanalaire présentait le même aspect histologique que la tumeur principale qui avait été étiquetée tumeur neuroendocrine bien différenciée de grade 2 de la classification OMS 2010 des TNE digestives (Figure 5). Devant cette découverte histologique, un bilan biologique (dosage de la sérotonine (5HT), de l'acide 5-hydroxyindole acétique (5HIAA), chromogranine A (CgA)) et un octréoscan avaient été réalisés à la recherche d'une autre localisation digestive et sont revenus négatifs signant la localisation hépatique primitive de cette tumeur. Le suivi de la patiente, après 2 ans et ½, ne note aucune récidive.

## Discussion

Les tumeurs neuroendocrines primitives du foie (TNE I^ve^) sont rares et représentent 0,3% de l'ensemble des tumeurs neuroendocrines qui, dans 67,5% des cas, naissent dans le tube digestif [1–3]; les lésions hépatiques correspondent souvent à des localisations secondaires de ces tumeurs [3]. L'expression clinique des TNE Ive du foie n'est pas spécifique, souvent liée à l'effet de masse qu'exerce la tumeur sur les structures de voisinage; le syndrome carcinoïde est rare, observé dans 10% des cas, et révèle souvent une TNE du grêle avec métastases hépatiques [1–3]. Dans le cas particulier de notre observation, la cholestase était liée au thrombus tumoral secondaire à l'extension de la lésion hépatique dans la VBP. Le diagnostic positif repose sur l’étude histologique de la biopsie de la lésion hépatique qui une fois révèle une tumeur neuroendocrine, un bilan biologique (dosage de 5T, 5HIAA, CgA) et morphologique (octréoscan, TDM, IRM, PET scan, endoscopie) doivent être réalisés à la recherche d'une autre localisation digestive avant de retenir le caractère primitif de la lésion hépatique. Chez notre patiente, l’étude histologique de la biopsie n’était pas contributive et les données de l'imagerie évoquait en premier lieu un processus tumoral des voies biliaires intra et extrahépatique sans extension à distance; en effet, le diagnostic de TNE I^ve^ du foie est difficile à poser sur les données de l'imagerie conventionnelle (échographie, TDM, IRM) où la lésion peut mimer un carcinome hépato-cellulaire, un cholangiocarcinome ou une métastase [2]. Le principal traitement des TNE^ives^ du foie reste l'exérèse chirurgicale dont l’étendue est conditionnée par le siège et la taille de la tumeur. Lorsque la résection est impossible (tumeur bilobaire, multicentrique), d'autres moyens thérapeutiques peuvent être utilisés: radiofréquence, embolisation sélective de l'artère hépatique, chimiothérapie régional ou systémique et les perfusions intraveineuses d'analogues de la somatostatine à visée symptomatique [4]. Un taux de survie de 74% à 5 ans après résection et un taux de récidive de 18% ont été rapportés, le suivi de notre patiente après 2 ans et demi de la résection ne montre aucune récidive.

## Conclusion

La rareté des tumeurs neuroendocrines primitives du foie et leur caractère clinique non spécifique font qu'elles sont rarement évoquées en première intention devant une lésion hépatique isolée et le diagnostic est souvent porté sur l'examen histologique de la biopsie de la tumeur ou de la pièce de résection chirurgicale.
